# Aetiological factors in molar incisor hypomineralisation: a case-control study from Salamanca, Spain

**DOI:** 10.1186/s13052-025-01972-2

**Published:** 2025-04-27

**Authors:** Johanna Muñoz, Alfonso Alvarado-Lorenzo, Laura Criado-Pérez, Laura Antonio-Zancajo, Marta Muñoz-Bruguier, Adrián Curto

**Affiliations:** Department of Surgery, Faculty of Medicine, University of Salamanca, Salamanca, Spain

**Keywords:** Children, Molar-incisor hypomineralisation, Paediatric dentistry

## Abstract

**Background:**

Molar incisor hypomineralisation (MIH) is a developmental dental condition that causes defects in the enamel of the first molars and permanent incisors. The aim of the present study was to assess possible causal correlations between the mother-child dyad medical history and MIH.

**Methods:**

An observational, retrospective, case‒control pilot study was carried out at the Dental Clinic of the University of Salamanca. This study was conducted between November 2023 and May 2024. Data on potential aetiological factors were collected through personal interviews, and the children’s parents were asked aetiological questions. Statistical analysis was performed with Student’s t test and the chi-square test.

**Results:**

A total of 140 children were enrolled in the study. The case group included 70 children with MIH (31 boys and 39 girls; mean age: 9.1 ± 2.32 years), while the control group comprised 70 children without MIH (32 boys and 38 girls; mean age: 9.57 ± 3.09 years). Among the factors assessed, maternal drug allergies during pregnancy and childhood asthma were identified as potential aetiological contributors to MIH, both showing statistically significant associations (*p* < 0.01).

**Conclusions:**

Within the limitations of this pilot case-control study, a potential association was observed between MIH and both maternal drug allergies during pregnancy and childhood asthma. These findings support the need for further investigation into prenatal and early-life factors that may contribute to enamel developmental disturbances. Larger prospective studies are recommended to confirm these associations and better understand the underlying mechanisms.

## Background

The term “molar incisor hypomineralisation” (MIH) was defined in 2003 by the European Academy of Paediatric Dentistry (EAPD). The first definition of MIH was in 2001. MIH is a dental pathology that involves qualitative enamel defects. This dental condition is characterised by the presence of hypomineralised enamel. This pathology affects at least the first permanent molars and can also affect permanent incisors [1, 2].

Clinically, MIH defects may appear as well-defined white, yellow, or brown opacities. These defects make the enamel more porous and susceptible to fractures, leading to a greater risk of coronary destruction of the teeth and premature tooth loss, with consequent negative repercussions on the oral health status of paediatric patients [3].

The aetiology of MIH is unknown. MIH studies describe a multifactorial etiology, in which there are genetic and environmental factors. Peri- and postnatal etiological factors are those that may be the most involved in its etiology [1–5]. The difference in the prevalence of MIH by geographical area also suggests some influence of genetics on its etiology [3, 6, 7]. Amongst the genetic factors, multiple polymorphisms in genes have been described related to amelogenesis, while different gene interactions are associated with an increased susceptibility in children to develop MIH [8, 9]. Different systemic factors can influence the formation of MIH lesions during the different stages of tooth development. It has been suggested, for example, that the type of delivery, low birth weight, the presence of fever during early childhood, and the consumption of antibiotics are factors that may be related to the appearance of MIH [10–12].

Both systemic and environmental factors during the prenatal period to the first three years of life can influence the development of this dental pathology. There is consensus in the scientific literature that children with a worse general health status in the first three years of life are more likely to develop MIH in the immediate future [13, 14].

There is great heterogeneity in the prevalence of MIH; depending on different published studies, the prevalence of MIH varies between 3% and 40% in the population of children between 6 and 12 years old. This disparity in the prevalence can be explained by the difference in the diagnostic criteria used in the different studies or by the sociodemographic characteristics of the populations studied. This high prevalence makes MIH a major oral public health problem in children [15].

Different authors have concluded that the prevalence of MIH is greater in girls than in boys [16–19]. It has also been observed that the prevalence of MIH in the upper teeth is greater than that in the lower teeth [18, 19].

MIH can cause serious dental problems such as dental hypersensitivity, chewing difficulties, pain and aesthetic problems in children [2, 20]. In severe cases, MIH can lead to enamel fracture and tooth loss. Studies show an increase in the prevalence of caries in patients with MIH. As the severity of MIH increases, DMF-T (decayed-missing-filled teeth index for permanent teeth) and DMF-S (decayed-missing-filled surfaces index for permanent teeth) index increase [21–23]. Early preventive intervention in patients affected by MIH is essential to reduce the severity of enamel lesions, the incidence of caries, and the repercussions on dentition [24, 25].

This condition has a negative impact on children’s oral quality of life. The physical and functional components of children with MIH are mainly affected by hypersensitivity, which makes daily oral hygiene and even correct nutrition difficult [26]. The possible premature tooth loss due to severe MIH affects occlusion and the need for orthodontic treatment in patients [26, 27].

The psychosocial burden of MIH has a great impact on paediatric patients. A child who is experiencing discomfort and/or chronic dental pain will have difficulty concentrating on their daily activities, which will have a negative impact on their schooling [26, 28–30].

MIH has a greater impact on oral quality of life in girls than in boys, although there are currently limited studies evaluating the influence of MIH on oral quality of life in the paediatric population [26, 28, 30]. A child’s emotional state can also be affected by the severity of the MIH lesions as well as the child’s age since dental aesthetics also play a key role [3, 31].

Scientific evidence is limited with respect to the analysis of the possible systemic and environmental aetiological factors that can influence the development of this dental pathology [32].

The objective of this retrospective, case‒control pilot study was to analyse the possible aetiological factors of MIH related to maternal health during pregnancy, childbirth conditions and the medical history in early childhood in a population of children in Salamanca (Spain).

## Methods

### Study design

This retrospective, case‒control pilot study was carried out at the Dental Clinic of the University of Salamanca. This study was conducted between November 2023 and May 2024 with a total sample of 140 participants.

Approval from the Research Ethics Committee of the University of Salamanca was obtained (protocol number 1077 and date or approval 11/27/2023). This project followed the guidelines established by the Declaration of Helsinki for research involving humans, as well as the Strengthening the Reporting of Observational Studies in Epidemiology (STROBE) guidelines for the conduct of observational studies. Participants were informed about the study procedures. Since the participants were minors, the consent of their parents or legal guardians was needed.

To identify patients with MIH, this study used the diagnostic criteria defined by the EAPD in 2021. The EAPD diagnostic criteria for MIH indicate that at least one permanent first molar must be affected for a diagnosis of MIH. One to four permanent first molars can be affected. Simultaneously, the permanent incisors may be affected [33, 34].

The oral examination of each patient was performed by a single examiner (A.C.). The examiner of this study is trained and experienced in the field of paediatric dentistry. As a dentist with training and experience in the field of pediatric dentistry, the problems that can arise from the performance of tasks by unqualified personnel and by different professionals were eliminated. The participants were grouped into two study groups: a case group (patients diagnosed with MIH) (*n* = 70) and a control group (patients without MIH) (*n* = 70).

### Eligibility criteria for participants

The inclusion and exclusion criteria were as follows:

Case group:

Inclusion criteria:

- Patients aged between 5 and 14 years.
- Patients whose first permanent molars had erupted.
- Patients diagnosed with MIH.

Exclusion criteria:

- Patients with significant physical and/or mental difficulties that hindered oral examination.
- Patients with defects in enamel development other than those related to MIH.
- Patients who were receiving orthopaedic and/or orthodontic treatment.
- Patients whose parents or legal guardians refused to participate in the study.

Control Group:

Inclusion criteria:

- Patients aged between 5 and 14 years.
- Patients whose first permanent molars had erupted.
- Patients without MIH.

Exclusion criteria:

- Patients with significant physical and/or mental difficulties that hindered oral examination.
- Patients with defects in enamel development other than those related to MIH.
- Patients who were receiving orthopaedic and/or orthodontic treatment.
- Patients whose parents or legal guardians refused to participate in the study.

### Analysis of the aetiological factors of MIH

In order to collect data on mother-infant dyad medical history during the perinatal life, a questionnaire was used, focusing on three areas of analysis, based on previous scientific studies [13, 14, 18, 32]. The questionnaire consisted of different items evaluating variables related to maternal health during pregnancy, childbirth conditions and the patients’ medical history. This questionnaire was completed with the children’s mothers at the same appointment at which the oral examination was performed.

A pilot test was carried out to evaluate the clarity of the questionnaire items; for this test, a convenience sample of 30 parents of patients not included in the study was randomly selected. Items that parents found difficult to answer and/or confusing were reviewed. After this test, the final questionnaire was developed. Factors related to maternal during pregnancy, the use of certain supplementation/supplements (folic acid, vitamin D, vitamin B12, iron, etc.) (for at least one week), and the presence of certain pathologies during pregnancy (high blood pressure, diabetes, or drug allergies) were analysed. In relation to the consumption of certain supplementation/supplements, it was considered that their consumption was for at least one month in pregnancy, while those consumed for less than one month were not considered. Smoking was considered as a study factor when the consumption was at least two cigarettes a day. Alcohol intake was considered valid when the mother consumed at least three units of alcohol per week. For variables related to childbirth, the duration of pregnancy, type of delivery, duration of delivery and infant weight at birth were analysed. The factors influencing MIH development in relation to the children’s medical history were breastfeeding status, asthma (diagnosed up to 6 years of age at most) and corticosteroid use (duration of use greater than one week) were considered.

### Statistical analysis

The data were analysed with the SPSS 28.0 software package. Student’s t test and the chi-square test were used. The significance level was set at *p* = 0.05, and any value less than or equal to 0.05 was considered to indicate statistical significance.

## Results

### Baseline characteristics of the population

A total sample of 140 participants aged between 5 and 14 years (median 9 years), with a mean age of 9.34 (± 2.73) years, was analysed. The participants in the study were divided into two groups: the case group (MIH group) (*n* = 70) and the control group (*n* = 70). In the case group, the mean age was 9.10 (± 2.32) years, and in the control group, it was 9.57 (± 3.09) years. The study groups were equal with respect to participant sex and age (Table 1).

**Table 1:** Sociodemographic characteristics of the sample

		Total sample (*n* = 140)	Case group (*n* = 70)	Control group (*n* = 70)	Student’s t test	*p* value	
Mean age (years) (SD)		9.34 (± 2.73)	9.10 (± 2.32)	9.57 (± 3.09)	1.02		0.309^NS^
Sex					Chi-squared		p value
	Boy	45.0% (63)	44.3% (31)	45.7% (32)	Chi^2^ = 0.03 (ns)		0.865^NS^
	Girl	55.0% (77)	55.7% (39)	54.3% (38)			

NS = Not significant (*p* > 0.05)

### Analysis of variables related to maternal health during pregnancy

In the analysis of the variables related to maternal health during pregnancy, it was observed that for most of the factors analysed, there was no statistical significance (*p* > 0.05), except the drug allergy factor (*p* < 0.01).

The analysis of maternal drug allergies during pregnancy revealed a statistically significant difference because all mothers with drug allergies were in the case group. The difference was generated due to the small number of patients with this factor (only *n* = 7); therefore, despite the statistical significance, this result must be considered with caution, and this study recommends analysing it in future studies with larger sample sizes (Table 2).

**Table 2:** Differences in variables related to maternal health during pregnancy between the study groups

Factor	Percentage (frequency)			Chi-squared test	
	Total sample (*n* = 140)	Case group (*n* = 70)	Control group (*n* = 70)	Statistical	*p* value
Folic acid consumption	84.3% (118)	88.6% (62)	80.0% (56)	1.94	0.164^NS^
Vitamin D consumption	22.1% (31)	25.7% (18)	18.6% (13)	1.04	0.309^NS^
Vitamin B12 consumption	14.3% (20)	17.1% (12)	11.4% (8)	0.93	0.334^NS^
Iron consumption	10.7% (15)	12.9% (9)	8.6% (6)	0.67	0.412^NS^
Levothyroxine use	8.6% (12)	10.0% (7)	7.1% (5)	0.36	0.546^NS^
Smoking	11.4% (16)	15.7% (11)	7.1% (5)	2.54	0.111^NS^
Viral infections and/or fever episodes (> 38 degrees Celsius)	7.9% (11)	11.4% (8)	4.3% (3)	2.47	0.116^NS^
Arterial Hypertension	12.9% (18)	15.7% (11)	10.0% (7)	1.02	0.313^NS^
Gestational diabetes	19.3% (27)	24.3% (17)	14.3% (10)	2.25	0.134^NS^
Urinary tract infection (last trimester of pregnancy)	12.9% (18)	15.7% (11)	10.0% (7)	1.02	0.313^NS^
Drug allergies	5.0% (7)	10.0% (7)	0.0% (0)	7.37	0.007^**^

NS = Not significant (*p* > 0.05); ** = Highly significant (*p* < 0.01).

### Analysis of variables related to childbirth

In the study of variables related to childbirth (Table 3), no statistical significance was detected. When analysing the weight of the newborns, it was observed that there may be a statistically significant relationship (*p* = 0.058): newborns with a weight less than 3 kg could be at risk of future MIH. It can be assumed that, upon analysing a larger population, the relationship between newborn birth weight and the risk of MIH could be confirmed.

**Table 3:** Differences in variables related to childbirth between the study groups

Factor		Percentage (frequency)			Chi-squared test	
		Total sample (*n* = 140)	Case group (*n* = 70)	Control group (*n* = 70)	Statistical	*p* value
Duration of pregnancy					1.56	0.669^NS^
	28–31 weeks	5.7% (8)	4.3% (3)	7.1% (5)		
	32–34 weeks	20.0% (28)	21.4% (15)	18.6% (13)		
	35–37 weeks	66.4% (93)	64.3% (45)	68.6% (48)		
	> 38 weeks	7.9% (11)	10.0% (7)	5.7% (4)		
Type of Delivery					0.76	0.384^NS^
	Vaginal	62.1% (87)	65.7% (46)	58.6% (41)		
	C-Section	37.9% (53)	34.3% (24)	41.4% (29)		
Duration of labour					0.04	0.981^NS^
	< 4 h	55.0% (77)	55.7% (39)	54.3% (38)		
	Between 4–8 h	17.1% (24)	17.1% (12)	17.1% (12)		
	> 8 h	27.9% (39)	27.1% (19)	28.6% (20)		
Newborn birth weight					3.58	0.058^NS^
	< 3 kg	40.7% (57)	48.6% (34)	32.9% (23)		
	> 3 kg	59.3% (83)	51.4% (35)	67.1% (47)		

NS = Not significant (*p* > 0.05)

### Analysis of variables related to the children’s medical history

The possible relationships among factors related to the medical history of children with MIH were also evaluated. The results revealed a statistically significant relationship (*p* < 0.01) between the presence of MIH and childhood asthma (Table 4).

**Table 4:** Differences in variables related to the children’s medical history between study groups

Factor		Percentage (frequency)			Chi-squared test	
		Total sample (*n* = 140)	Case group (*n* = 70)	Control group (*n* = 70)	Statistical	*p* value
Breastfeeding status					2.98	0.396^NS^
	No breastfeeding	15.7% (22)	18.6% (13)	12.9% (9)		
	1–6 months	28.6% (40)	25.7% (18)	31.4% (22)		
	6–12 months	28.6% (40)	32.9% (23)	24.3% (17)		
	> 12 months	27.1% (38)	22.9% (16)	31.4% (22)		
Asthma		10.7% (15)	20.0% (14)	1.4% (1)	12.62	0.001^**^
Use of corticosteroids		7.9% (11)	11.4% (8)	4.3% (3)	2.47	0.116^NS^

NS = Not significant (*p* > 0.05); ** = Highly significant (*p* < 0.01).

## Discussion

The objective was to find any correlations between specific and selected data from maternal and infant perinatal medical history and the diagnosis of MIH/or and the MIH phenotype, including maternal health during pregnancy, pregnancy and childbirth conditions, and child health. This study analysed the possible aetiological factors in a population of children in Salamanca (Spain) aged between 5 and 14 years.

Due to the lack of previous studies on the possible statistical association between different maternal and patient factors in the occurrence of MIH in the region of Salamanca (Spain), the sample size was set at a preliminary level of 140 participants in this pilot study. However, it should be noted that there are published studies with smaller sample sizes than the one used in this study [14, 35, 36].

According to the scientific literature, the reported prevalence rates of MIH vary from 3 to 40% in the paediatric population aged between 6 and 12 years, which was the age range analysed in this study [15, 37, 38].

At present, based on the scientific literature, the aetiological mechanism of MIH is not known. Systemic and environmental factors act as possible aetiological variables [3, 13, 14, 32, 38–42]. The possible genetic origin of MIH [13, 40] has also been described.

This study used a convenience sample of patients who visited the dental clinic of the University of Salamanca for dental assistance. The sample analysed was homogeneous with respect to participant sex and age.

The information related to the questionnaire developed in this study was obtained only from the children’s mothers. For the development of this questionnaire, the aetiological factors described in the scientific literature were considered [13, 14, 18, 32, 39, 40, 43].

### Variables related to maternal health during pregnancy

In this study, it was observed that the presence of maternal drug allergies during pregnancy had a statistically significant influence on patients with MIH. Other previous studies have revealed that the presence of urinary tract infections during pregnancy [37, 44] and the use of antibiotics during pregnancy [44, 45] are associated with an increase in the incidence of MIH.

In this study, vitamin D intake during gestation was not found to be statistically significantly related to MIH. There are studies in the literature that have evaluated the association between vitamin D supplementation during gestation and the risk of early childhood enamel defects in children. Published studies report that vitamin D supplementation during pregnancy may be a protective and preventive intervention against the development of enamel defects and MIH in children [46–48].

### Variables related to childbirth

In relation to the possible aetiological factors related to childbirth and directly related to MIH, this study concluded that all analysed causes with respect to childbirth had a statistically significant relationship with MIH.

Other authors reported that the type of delivery (vaginally delivery or caesarean section) could be a possible aetiological factor affecting the incidence of MIH. These authors concluded that delivery by caesarean section is directly proportional to the development of MIH [37, 44].

### Variables related to the children’s medical history

This study revealed that patients with a history of asthma were more predisposed to develop MIH (*p* < 0.01). The results described in this study are consistent with those described by other authors [43, 49]. However, regarding medical history, there is a certain disparity among the different published studies: some authors concluded that the presence of certain diseases during childhood, such as chickenpox [37, 49], atopic dermatitis [50], neonatal jaundice [51], rubella [49], mumps [49], and tonsillitis [43], or the use of drugs for long periods during childhood [37, 43, 44, 52], increases the risk of MIH.

Therefore, and as described above, there is disparity between the conclusions reported in previously published studies based on the possible aetiological factors related to MIH.

### Limitations

Sample size composition is a strong limitation in this study; although it should be noted that this study was a pilot study. One of the main limitations of this study was the use of a self-designed questionnaire to evaluate the possible aetiological factors of MIH since the use of an unvalidated questionnaire can limit the reliability of study results. Another limitation was that the population analysed was from a single geographical region. Due to these limitations, the results described in this study should be interpreted with caution. Due to the design and methodology of this study, we can only establish associations and not causes and effects. Future multicentre studies with larger sample sizes are necessary to better understand the aetiological factors of MIH. Previous studies were carried out considering the populations of specific regions; therefore, there is a need to carry out future studies that cover a wider population and analyse different regions. These future studies will be more convincing when the biological mechanisms that cause MIH are detected. There are different situations or factors that may be involved in the aetiology of MIH that cannot be controlled or predicted.

## Conclusions

- In relation to factors related to maternal health during pregnancy, in this study it was observed that drug allergies were directly proportional to the development of MIH (*p* < 0.01).
- Regarding the children’s health status, it was concluded that asthma had a statistically significant influence on the development of MIH (*p* < 0.01).
- Multicentre studies with larger sample sizes are needed to determine how to more accurately determine the aetiological factors of MIH related to maternal health during pregnancy, childbirth conditions and child health.

## Data Availability

The datasets generated during the current study are available from the corresponding author on reasonable request.

## Abbreviations

EAPD: European Academy of Paediatric Dentistry
MIH: Molar incisor hypomineralisation
